# Comparison of different timings of percutaneous coronary intervention in patients with transcatheter aortic valve implantation: a network meta-analysis

**DOI:** 10.3389/fcvm.2025.1596208

**Published:** 2025-08-01

**Authors:** Qi Wen, Jiuyu Yang, Guomin Xu, Da'an Wang

**Affiliations:** ^1^ Cardiovascular Medicine Department, Hulunbuir People's Hospital, Hulunbuir, Inner Mongolia, China; ^2^ Department of Cardiovascular and Structural Heart Disease, Inner Mongolia Forestry General Hospital, Yakeshi, Inner Mongolia, China

**Keywords:** CAD, TAVI, PCI, mortality, network meta-analysis

## Abstract

**Background:**

The combination of selective percutaneous coronary intervention **(**PCI) and transcatheter aortic valve implantation (TAVI) is a safe and feasible therapy and has become our preferred treatment option for patients with severe aortic stenosis and high-risk coronary heart disease. However, the timing of staged PCI is uncertain. The purpose of this meta-analysis is to compare the benefits and risks of TAVI alone, PCI before TAVI, simultaneous TAVI and PCI, and PCI after TAVI in TAVI patients, and to provide guidance for clinical decision-making on the timing of PCI in TAVI patients.

**Methods:**

We searched Pubmed, Embase, the Cochrane Library and Web of Science as of April 2025. By employing Bayesian network meta-analysis, with the aid of R software (V4.3.2) and in combination with Stata (V15), the analysis included outcomes such as all-cause mortality, cardiovascular mortality, stroke, bleeding and myocardial infarction (MI). Pooled analysis was performed utilizing risk ratios (RR) and 95% confidence intervals (CI).

**Results:**

A total of 13 studies involving 304,181 patients were included in the analysis. The research findings showed that the application of TAVI alone significantly reduced the all-cause mortality compared to PCI after TAVI (RR = 0.35, 95% CrI: 0.13, 0.88), and the lowest all-cause mortality rate was observed in the cumulative ranking (SUCRA = 75.89%). Compared with PCI after TAVI (RR = 0.57, 95% CrI: 0.41, 0.79) and TAVIplus PCI (RR = 0.72, 95% CrI: 0.54, 0.97), PCI before TAVI significantly reduced cardiovascular mortality and was found the lowest cardiovascular mortality in the cumulative ranking (SUCRA = 98.37%). In comparison to TAVIplus PCI (RR = 0.44, 95% CrI: 0.27, 0.71), PCI after TAVI significantly reduced the stroke rate and found the lowest stroke rate in the cumulative ranking (SUCRA = 97.21%). The application of TAVI alone significantly reduced the bleeding rate compared to TAVIplusPCI (RR = 0.61, 95% CrI: 0.60, 0.62), and the lowest bleeding rate was observed in the cumulative ranking (SUCRA = 88.14%). Compared with PCI before TAVI (RR = 0.12, 95% CrI: 0.04, 0.29) and TAVI (RR = 0.21, 95% CrI: 0.12, 0.34), TAVIplusPCI significantly reduced the incidence of myocardial infarction and was found the lowest incidence of myocardial infarction in the cumulative ranking (SUCRA = 96.44%).

**Conclusion:**

The timing of application of TAVI combined with PCI affects mortality and the incidence of cardiovascular events. Among them, PCI after TAVI may effectively reduce all-cause mortality, cardiovascular mortality, and stroke, but the interval between the two procedures remains uncertain. Future studies should investigate the optimal interval between PCI and TAVI to maximize clinical benefits.

**Systematic Review Registration:**

https://www.crd.york.ac.uk/PROSPERO/, PROSPERO.

## 1 Introduction

Aortic stenosis (AS) is the most common severe valvular heart disease (VHD) (1), especially in European and American countries, and its incidence is also rapidly increasing (2). The incidence of coronary artery disease (CAD) in severe AS patients has increased to 75% (3). According to an epidemiological survey, 59.3% of severe AS patients may die within 5 years if they do not receive treatment aggressively (4). Studies have found that risk factors of aortic sclerosis and stenosis are similar to those of atherosclerosis (2, 4), including smoking, hypertension, diabetes and levels of high and low-density lipoprotein cholesterol (LDL-C).

Severe calcific AS is often accompanied by obstructive CAD (5, 6). European and American guidelines recommend that, if there are indications for surgical or interventional treatment, both CAD and AS should be treated simultaneously (7, 8). Transcatheter aortic valve replacement (TAVR) was traditionally the sole treatment option for AS. However, transcatheter aortic valve implantation (TAVI) is now a safe and effective alternative (9). Although TAVI, initially limited to patients deemed unsuitable for surgery, is now used in those with intermediate-to-high surgical risk and offers symptomatic relief, it carries an increased risk of major vascular complications within 30 days and at 1 year (10, 11). International guidelines further recommend (12, 13) that for patients with proximal coronary stenosis ≥70% who plan to receive TAVI, timely PCI should be considered. The study by Abdel-wahab, M found that (14) patients undergoing TAVI combined with PCI showed significant improvement in clinical symptoms after surgery and over 6 months. However, the optimal timing for PCI intervention remains unclear. Nicolas M Van Mieghem et al. found that (15) elderly patients with severe AS receiving TAVI treatment showed a higher mortality after complete revascularization treatment. However, Yigal Abramowitz (16) and Guo Y (17) found that the combination of TAVI and PCI did not increase the risk of perioperative complications or all-cause mortality.

Although PCI combined with TAVI represents a safe and viable strategy, the optimal timing for PCI intervention has not been clearly established; specifically, whether it should be performed prior to, during, or following TAVI. Currently, numerous studies directly compare PCI combined with TAVI to TAVI alone, but lack evaluation of different timings of PCI intervention. This study aims to perform network meta-analysis to compare the clinical outcomes of different timings of PCI intervention, providing more specific guidance on timing for TAVI patients who require PCI.

## 2 Methods

This study adhered to the Preferred Reporting Items for Systematic Reviews and Meta-Analyses (PRISMA) guidelines and its requirements for NMA (18). This meta-analysis was implemented according to the PRISMA Guidelines. The study protocol was registered in the International Prospective Register of Systematic Reviews (No.: CRD42023483587).

### 2.1 Search strategy

The literature in English was searched from the establishment of each database (PubMed, Embase, Cochrane Library, and Web of Science) to December 24, 2024. The retrieval was carried out by combining subject headings and text words, and the medical subject headings were as follows: TAVI, PCI, CAD, AS. The specific search strategy employed is documented in Supplementary Table S1. Additionally, a secondary search was performed to the references of published systematic reviews to ensure comprehensive literature coverage.

### 2.2 Inclusion and exclusion criteria

The inclusion criteria were as follows: (1) Study population: Patients received TAVI. (2) Intervention measures: TAVI alone, TAVI combined with PCI, which was divided into PCI before TAVI, simultaneous TAVI and PCI (TAVIplusPCI), and PCI after TAVI. (3) Study type: randomized controlled trials (RCTs) and cohort studies. (4) Outcome measures: Primary outcome: all-cause mortality, cardiovascular mortality; Secondary outcome: The event of stroke, bleeding and myocardial infarction. (5) There are no constraints on the quality of the research article.

The following studies were excluded: (1) Animal or cell experiments, case reports, scientific experimental plans, comments, letters, editorials, conference papers, etc.; (2) Articles with missing data or serious errors; (3) Duplicates; (4) Studies for which the full text could not be accessed; (5) There was no clear timing for PCI combined therapy.

### 2.3 Literature screening and data extraction

The retrieved literature was imported into EndNote. Two researchers independently screened the titles and abstracts of the articles based on the inclusion and exclusion criteria, followed by a full-text reading for a second screening. Any disagreements on the literature were resolved through discussion or consultation with a third researcher for reassessment. The two researchers independently extracted data information from the final included studies using Excel 2016, including the first author, year of publication, country, study type, intervention measures, sample size, age, body mass index (BMI), follow-up time, outcome extraction time, and outcome indicators.

### 2.4 Quality assessment

Articles meeting the above criteria were assessed according to the Newcastle-Ottawa Scale score (19). The NOS consisted of three aspects: selection, comparability, and outcome. The quality of the study was assessed as follows: low quality = 0–3; moderate quality = 4–6; and high quality = 7–9.

The included studies were assessed for bias using the Cochranerisk of bias tool (RoB2.0) (20) from 5 aspects: bias arising from the randomization process, bias due to deviations from the intended interventions, bias due to missing endpoint data, bias in measurement of the endpoints, and bias in the selection of the reported endpoints. For each study, two researchers independently made quality assessment judgments to categorize each of the five aspects as “low risk”, “high risk”, or “some concerns”. Discrepancies were resolved through discussion or consultation with a third researcher. The results of the assessments were presented in a risk of bias graphs.

### 2.5 Statistical analysis

The outcomes were displayed as risk ratio (RR) with 95% confidence intervals (CIs). In view of the heterogeneity between trials, the Bayesian hierarchical random-effects model was first fitted for multiple comparisons of different treatment options for comparison of different timings of PCI in patients with TAVI (21, 22). All the calculations and graphs were obtained using the R 4.4.2 software and Stata 15.1 software. Based on the theory of likelihood function and some prior assumptions, Markov chain A Monte Carlo Markov Chain (MCMC) simulation was conducted employing Bayesian inference with R 4.4.2 software. The simulation comprised 500,000 iterations and 20,000 annealing steps to explore the posterior distributions of the nodes under investigation (22–25). The node-splitting method was used to evaluate local inconsistency for outcomes with closed loops. The relationships among the different treatments were presented as a network graph; meanwhile, a comparison-adjusted funnel plot was utilized to test for potential publication bias (26, 27). Moreover, we adopted the surface under the cumulative ranking (SUCRA) values to rank the examined treatments, and the SUCRA values ranged from 0–1. A higher SUCRA value indicated to a higher ranking for treatments (28, 29). A league table was generated to present the comparisons between each pair of interventions within each outcome.

## 3 Results

### 3.1 Literature retrieval and screening

**Figure 1:**
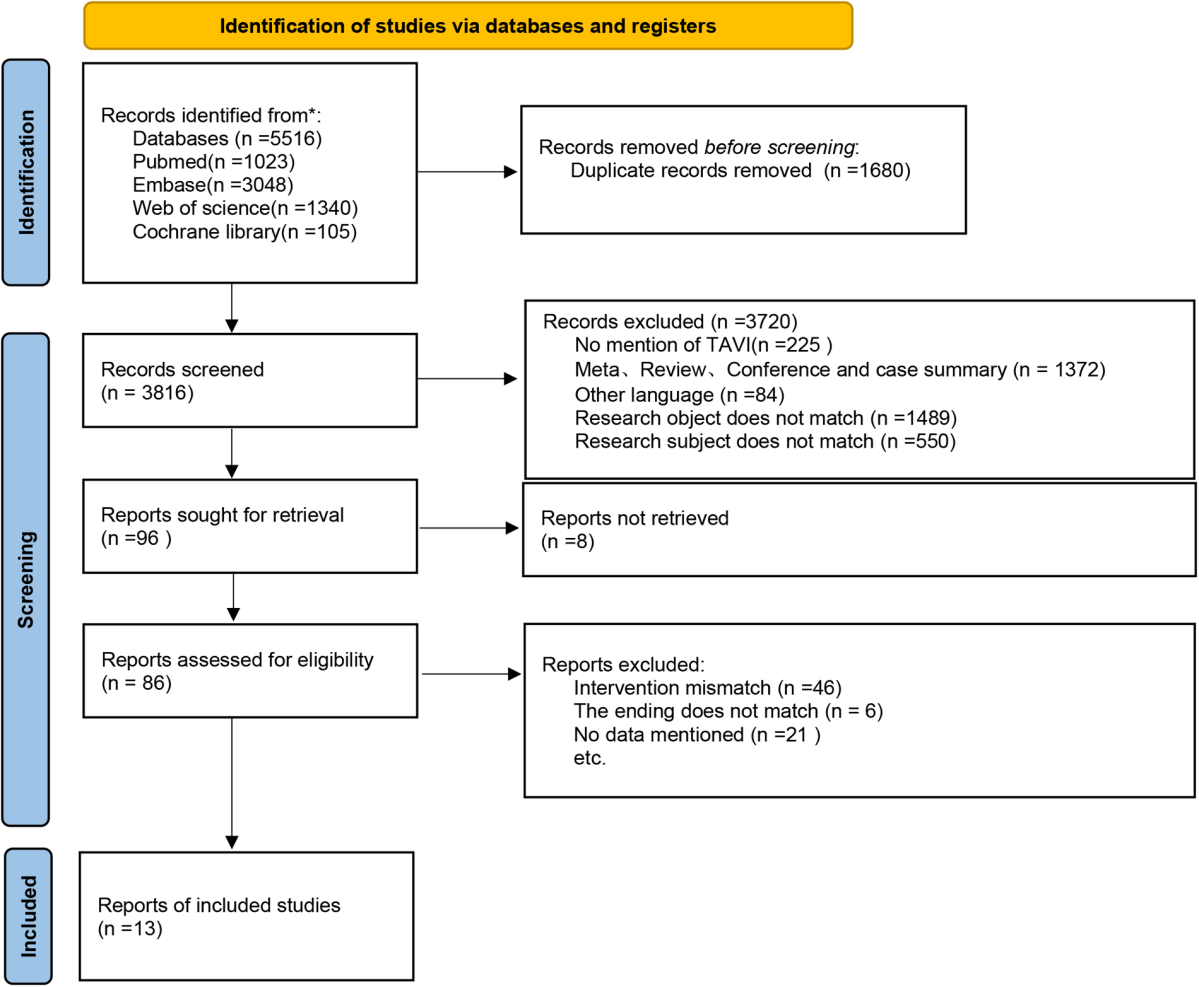
Flowchart.

A total of 5,516 articles were retrieved in the search, out of which 1,680 duplicates were excluded. After a preliminary review of titles and abstracts, 3,720 articles were further eliminated. The full texts of the remaining articles were reviewed, during which strict inclusion and exclusion criteria were applied. Finally, 13 articles were included. The detailed screening process is illustrated in Figure 1.

### 3.2 Basic characteristics and quality assessment of included studies

The 13 included studies were conducted in 7 countries, including Netherlands, Germany, the United States, the United Kingdom, Australia, Italy and Lebanon, involving a total of 304,181 patients. Of these, 12 studies reported the gender distribution, with 161,678 males and 152,503 females. The age of the participants ranged from 68.3–90.4 years. Basic characteristics of the included studies are presented in Supplementary Table S2.

All studies were cohort studies with a quality score of 7 or higher, indicating high-quality research (Supplementary Table S2).

### 3.3 Network analysis results

#### 3.3.1 Network diagram

**Figure 2:**
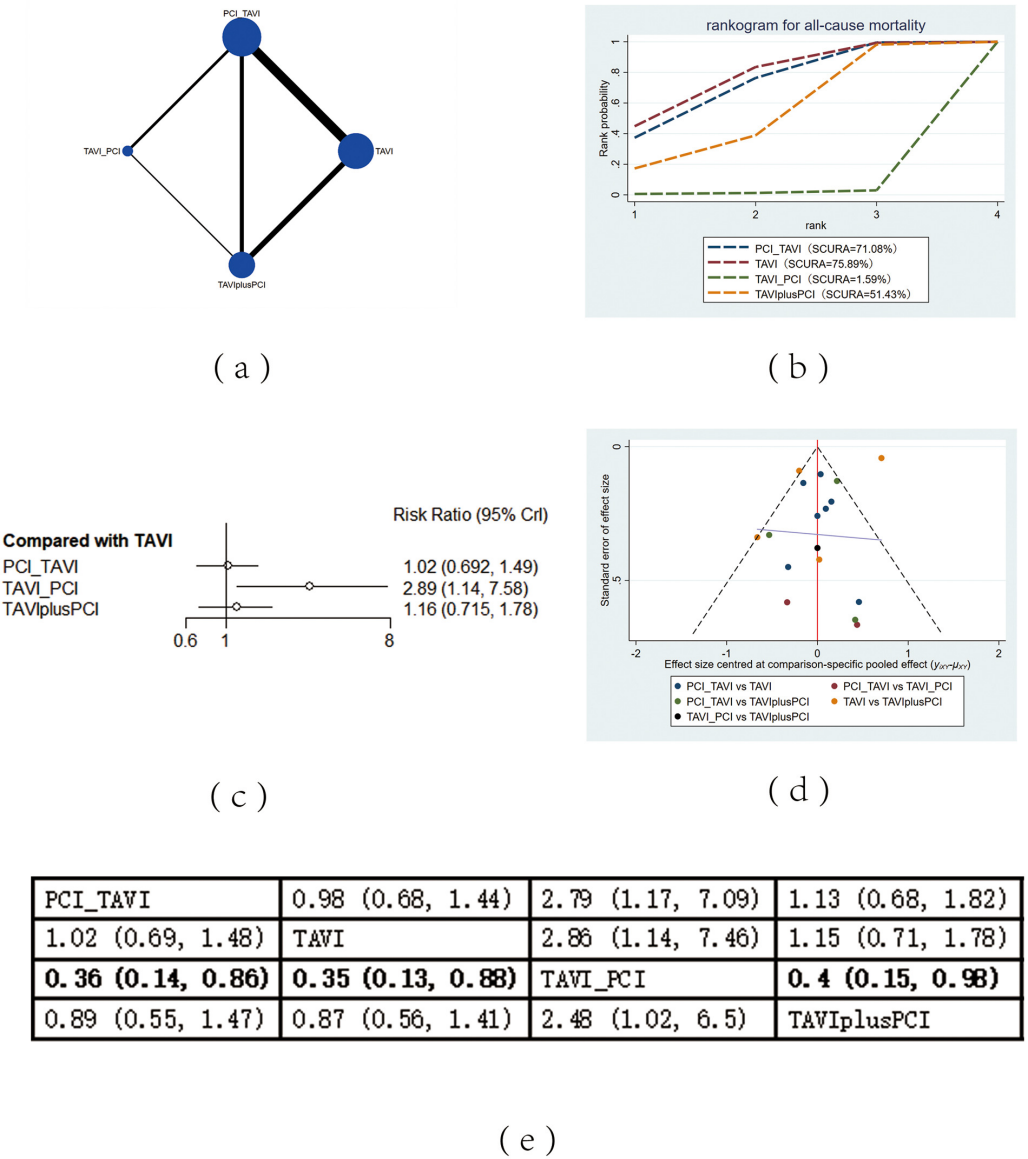
All-cause mortality. **(a)** Network diagram of all-cause mortality; **(b)** Line plot of all-cause mortality; **(c)** Forest plots of all-cause mortality; **(d)** Funnel plot of all-cause mortality; **(e)** League table of all-cause mortality.

**Figure 3:**
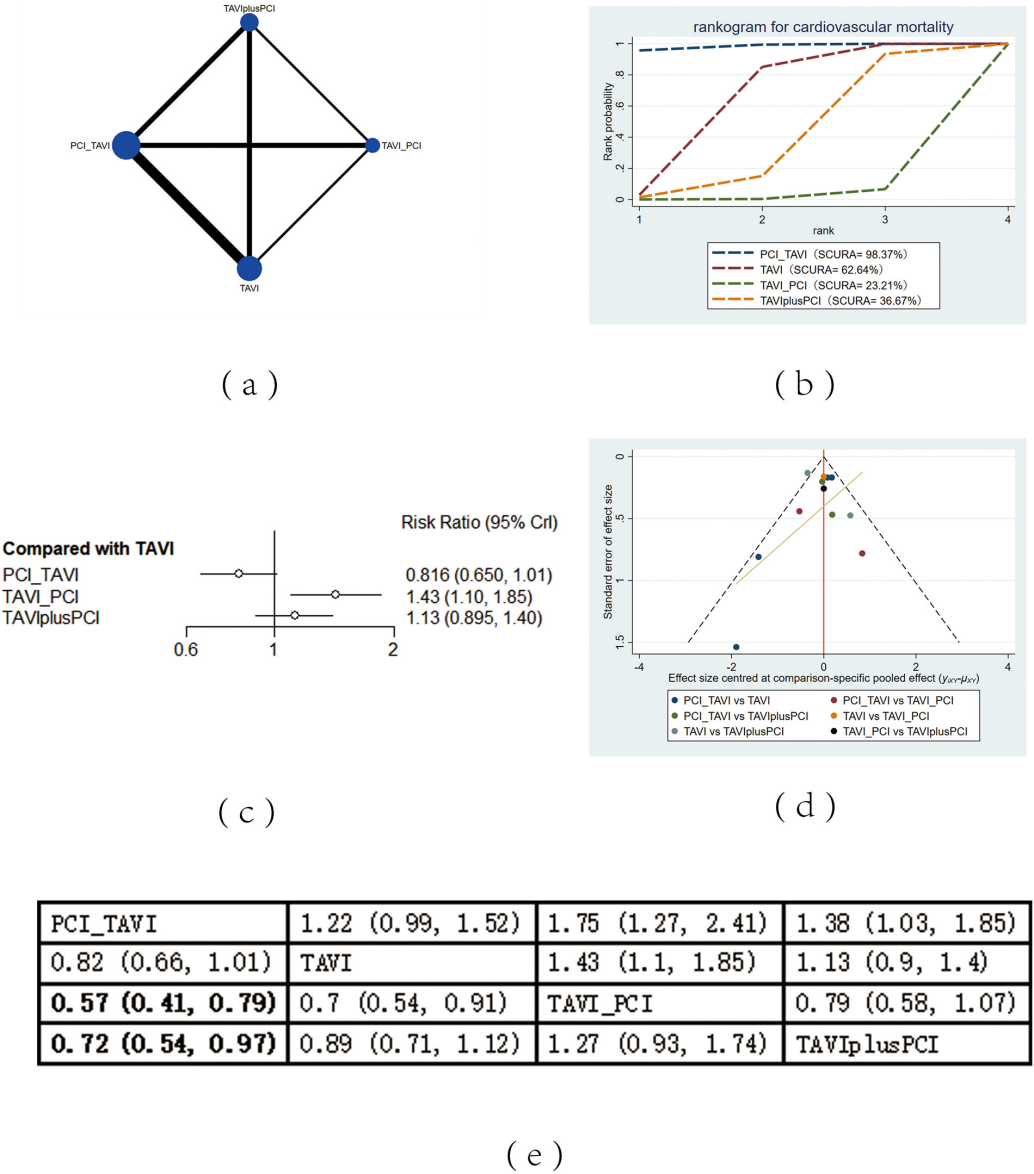
Cardiovascular mortality. **(a)** Network diagram of cardiovascular mortality; **(b)** Line plot of cardiovascular mortality; **(c)** Forest plot of cardiovascular mortality; **(d)** Funnel plot of cardiovascular mortality; **(e)** League table of cardiovascular mortality.

**Figure 4:**
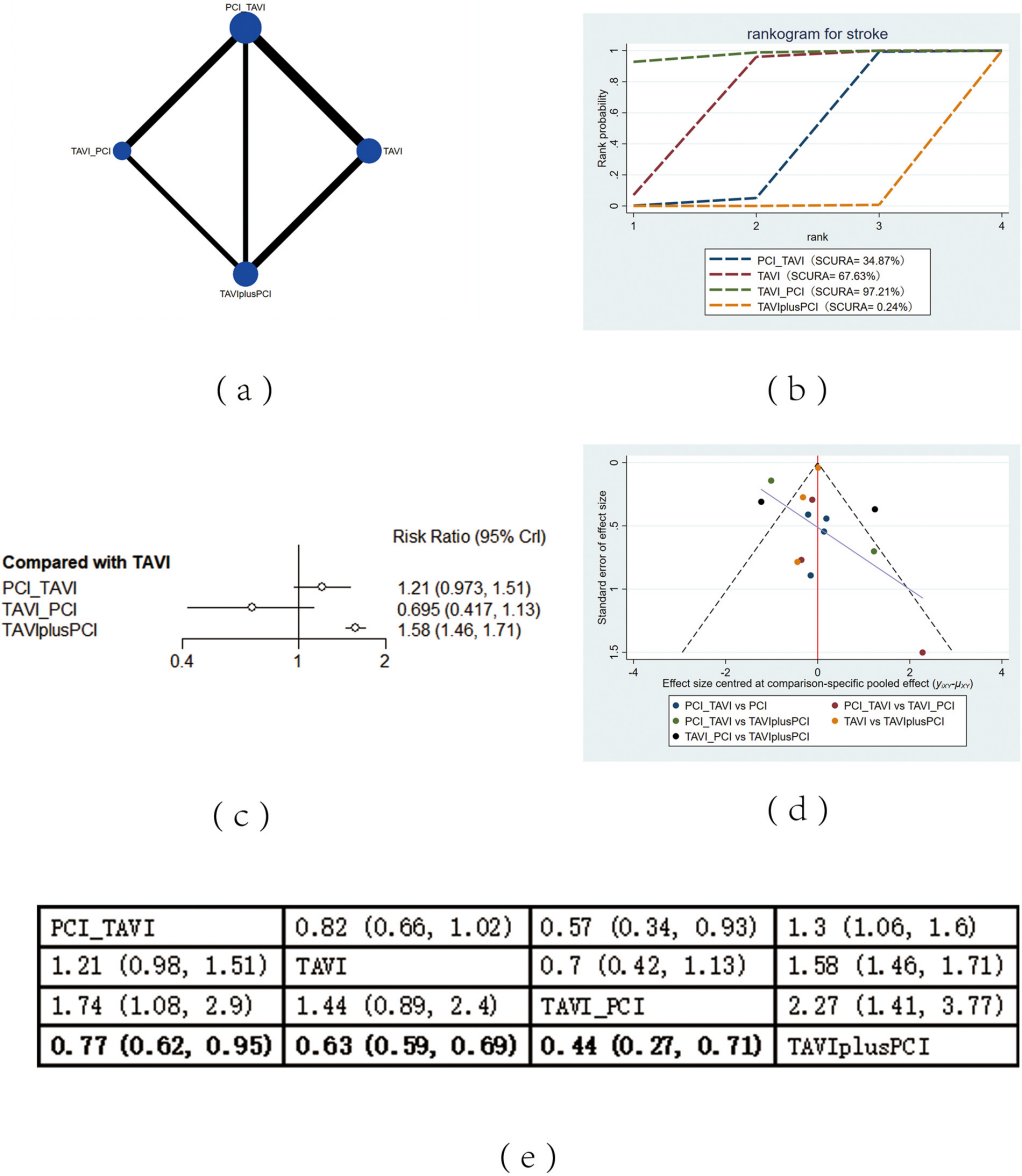
Stroke. **(a)** Network diagram of stroke; **(b)** Line plot of stroke; **(c)** Forest plot of stroke; **(d)** unnel plot of stroke; **(e)** League table of stroke.

**Figure 5:**
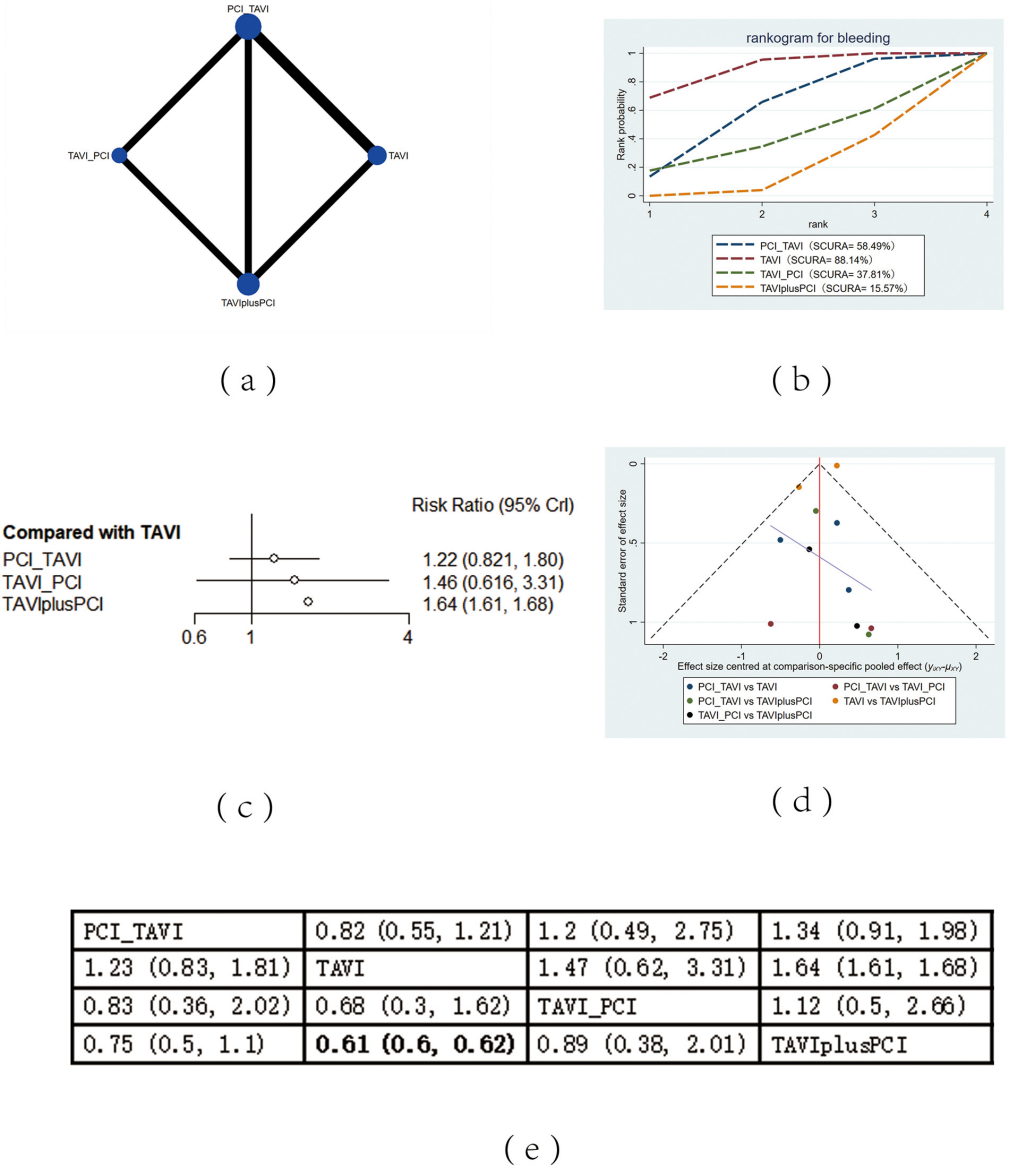
Bleeding. **(a)** Network diagram of bleeding; **(b)** Line plot of bleeding; **(c)** Forest plot of bleeding; **(d)** Funnel plot of bleeding; **(e)** League table of bleeding.

**Figure 6:**
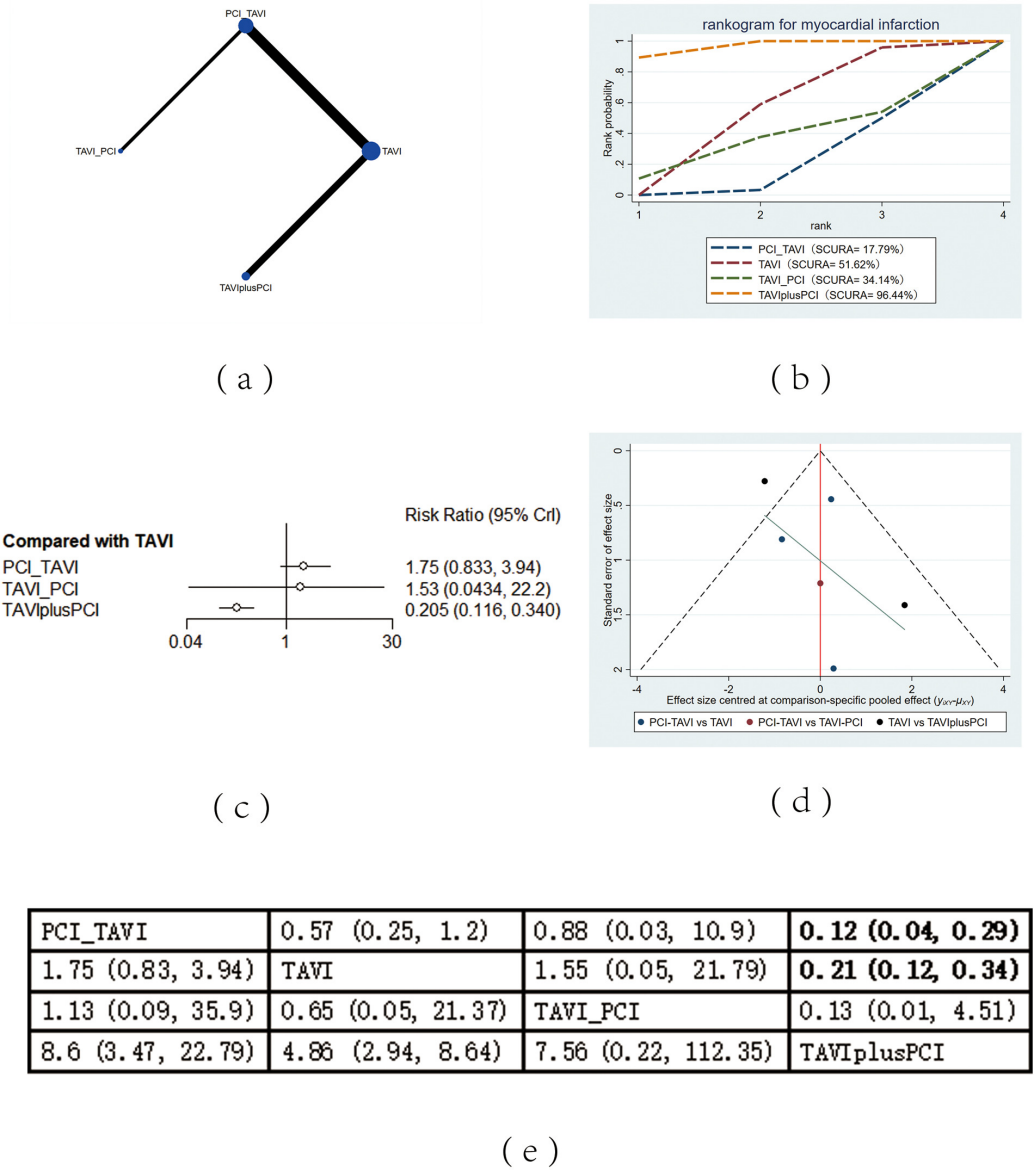
Myocardial infarction. **(a)** Network diagram of myocardial infarction; **(b)** Line plot of myocardial infarction; **(c)** Forest plot of myocardial infarction; **(d)** Funnel plot of myocardial infarction; **(e)** League table of myocardial infarction.

The included 13 studies covered 4 different intervention measures: TAVI, PCI before TAVI, PCI after TAVI, TAVIplusPCI. The network structure diagram illustrating the relationships among these different interventions is shown in Figures 2a, 3a, 4a, 5a, 6a. In the figure, the thickness of lines is proportional to the number of articles included in the pairwise comparisons, and the diameter of the circles is proportional to the number of participants included in the interventions. If the node-splitting method is used for analysis, the description is as follows: Node-splitting analysis will be conducted for outcomes with closed loops. All *P* values greater than 0.05 indicate no evidence of local inconsistency.

#### 3.3.2 All-cause mortality

All-cause mortality was reported in 13 studies. The results showed that compared with PCI after TAVI, PCI before TAVI (RR = 0.36, 95% CI: 0.14, 0.86), the application of TAVI alone (RR = 0.35, 95% CI: 0.13, 0.88) and TAVIplusPCI (RR = 0.4, 95% CI: 0.15, 0.98) effectively decreased all-cause mortality. According to the cumulative probability results based on SUCRA (Figure 2b), the all-cause mortality may be the lowest when TAVI (0.76) alone, PCI before TAVI (0.71), or PCI after TAVI (0.02) was applied.

#### 3.3.3 Cardiovascular mortality

Cardiovascular mortality was reported in 13 studies. The results showed that compared with PCI after TAVI (RR = 0.57, 95% CI: 0.41, 0.79) and TAVIPlusPCI (RR = 0.72, 95% CI: 0.54, 0.97), PCI before TAVI effectively decreased cardiovascular mortality. Compared with PCI after TAVI (RR = 0.7, 95% CI: 0.54, 0.91), the application of TAVI alone also effectively decreased cardiovascular mortality. According to the cumulative probability results based on SUCRA (Figure 3b), the cardiovascular mortality may be the lowest when PCI before TAVI (0.98), TAVI (0.62) alone, or PCI after TAVI (0.02) was applied.

#### 3.3.4 Stroke

The mortality rate of stroke was reported in 13 studies. The results showed that compared with TAVIplusPCI, the application of PCI before TAVI (RR = 0.77, 95% CI: 0.62, 0.95), TAVI alone (RR = 0.63, 95% CI: 0.59, 0.69) and PCI after TAVI (RR = 0.44, 95% CI: 0.27, 0.71) effectively reduced the mortality rate of stroke. Compared with PCI before TAVI, PCI after TAVI (RR = 0.57, 95% CI: 0.34, 0.93) also effectively decreased the mortality rate of stroke. According to the cumulative probability results based on SUCRA (Figure 4b), the mortality rate of stroke may be the lowest when PCI after TAVI (0.97), TAVI (0.68) alone, or PCIplusTAVI (0.002) was applied.

#### 3.3.5 Bleeding

The mortality rate of bleeding was reported in 13 studies. The results showed that compared with TAVIplus PCI, the application of TAVI alone (RR = 0.61, 95% CI: 0.6, 0.62) effectively decreased the mortality rate of bleeding. According to the cumulative probability results based on SUCRA (Figure 5b), the bleeding mortality may be the lowest when TAVI (0.88) alone, PCI before TAVI (0.58), or TAVIplusPCI (0.16) was applied.

#### 3.3.6 Myocardial infarction

The mortality rate of MI was reported in 13 studies. The results showed that compared with PCI before TAVI (RR = 0.12, 95% CI: 0.04, 0.29) and TAVI alone (RR = 0.21, 95% CI: 0.12, 0.34), TAVIplusPCI effectively reduced the mortality rate of MI. According to the cumulative probability results based on SUCRA (Figure 6b), the mortality of MI may be the lowest when TAVIplusPCI (0.96), TAVI (0.52) alone, or PCI before TAVI (0.18) was applied.

### 3.4 Heterogeneity and publication bias

Heterogeneity tests were performed for all outcomes, revealing that the majority of interventions had low to moderate heterogeneity. For detailed information, please refer to Supplementary Figures S1–S5 in the appendix. Funnel plots were used to evaluate the publication bias of all outcome indicators. The funnel plots for all-cause mortality (Figure 2d), cardiovascular events (Figure 3d), stroke (Figure 4d) outcomes and bleeding (Figure 5d) outcomes were symmetrical, indicating the absence of publication bias. The funnel plots for myocardial infarction were asymmetrical, suggesting that there might be certain publication bias.

## 4 Discussion

To our knowledge, this is the first meta-analysis (NMA) comparing the effectiveness and safety of different timings of PCI in TAVI. This NMA analyzed the most recent data from 13 cohort studies. All-cause mortality outcomes showed that TAVI alone was significantly superior to PCI after TAVI. Cardiovascular event outcomes indicated that PCI before TAVI effectively reduced cardiovascular mortality compared to PCI after TAVI. Compared with PCI after TAVI, TAVI alone significantly decreased cardiovascular mortality rates. The stroke outcome revealed that the stroke rate of PCI after TAVI was significantly lower than that of TAVIplus PCI. The bleeding outcome showed that compared with TAVIplusPCI, the application of TAVI alone effectively reduced the bleeding rate. The outcome of MI showed that TAVIplusPCI effectively reduced the mortality rate of MI compared to PCI before TAVI.

TAVI as a standalone procedure is no longer indicated for the treatment of patients with severe symptomatic aortic stenosis, as its adverse effects are exacerbated in those with concomitant CAD (30, 31). Therefore, many patients undergoing TAVI also require PCI (32–34). Previous trials have also shown that both PCI before TAVI and TAVI before PCI are safe procedures in patients with relatively low SYNTAX scores and few patients with multivessel disease (35). Performing revascularization prior to TAVI has become a widely adopted practice in most TAVI centers (36). In contrast to simultaneous TAVI and PCI, PCI before TAVI was not associated with any significant difference in 30-day mortality, severe/life-threatening bleeding, or major vascular complications (37). Furthermore, Montalto et al. reported that PCI before TAVI, compared to simultaneous TVAI and PCI, showed no differences in all-cause mortality, cardiovascular mortality, or the occurrence of stroke and myocardial infarction. These findings collectively support the safety of PCI before TAVI (38). The research findings showed that compared with PCI after TAVI, the application of PCI before TAVI, PCI alone and TAVIplusPCI effectively reduced all-cause mortality. The results from Gasparetto V et al. (39) revealed that 20.4% of CAD patients who underwent TAVI surgery did not experience any adverse events during PCI before TAVI. Mohamed Abdel-Wahab (14) and Abramowitz Y (16) focused on high-risk elder patients with severe CAD and severe AS, and found that PCI before TAVI was feasible and safe, and this combination therapy did not increase the risk of perioperative complications or all-cause mortality. The study by Tiffany Patterson et al. (40) showed that in terms of the 1-year all-cause mortality extraction outcome, there was no difference between PCI before TAVI and TAVI alone. They mainly focused on people with single coronary artery disease and the old population (with an average age of 84 years) in their research on ACTIVATION. However, we found some different conclusions in other studies. According to the study by Sachin S. Goel (41), PCI before TAVI did not increase the risk of short-term adverse outcomes. Meanwhile, the study by Rafael A Kotronias Rafail A Kotronias et al. (37) showed that PCI before TAVI did not have clinical advantages and might increase the 30-day mortality, while its drawback was the lack of stratification of CAD severity. Tobias Rheude et al. found that (42) the all-cause mortality rate and the composite endpoint incidence of patients receiving PCI after TAVI were significantly lower than those of patients receiving PCI before TAVI or TAVIplusPCI. Mattia Lunardi et al. (43) found that PCI after TAVI did not increase perioperative mortality and appeared to show a good trend in long-term outcomes. This advantage was only observed in the use of balloon valvuloplasty.

Our results for the outcome of MI showed that TAVIplusPCI effectively reduced the mortality rate of MI compared to PCI before TAVI and TAVI alone. Our results were inconsistent with those of Daniel P. Griese et al. (44). Regarding the outcome of MI, his results showed that patients who underwent elective PCI had a higher mortality rate compared to those who received TAVI alone. Regardless of synchronous or staged PCI strategies, the perioperative mortality rate increased threefold. However, his research mainly focused on treating a single coronary artery, while our study did not provide a detailed differentiation of the branch number of vascular lesions, which may be the reason for the differences in results. The study by Johannes Blumenstein (45) suggested that the timing of PCI had no impact on mortality rate in MI outcomes. According to the study by Daniel P. Griese et al. (44), patients who underwent TAVIplusPCI had a higher incidence of myocardial infarction, and one mechanism for the increased mortality rate in the PCI group was the accumulation of myocardial infarction (especially during the perioperative period). This is also consistent with our conclusion that TAVIplusPCI effectively reduces the mortality rate of MI. In a study conducted by Mohamed Abdel-Wahab on small groups (14), no patients experienced perioperative myocardial infarction or stroke, with a 30-day mortality rate of 7.1%. In a practical study conducted by Nunes RAB et al. (46) on patients with CAD and severe AS, patients who received TAVI alone had no difference in 5-year mortality and the probability of ischemic stroke compared to those who received PCI before TAVI or during TAVI. In contrast to our study demonstrating the effectiveness of PCI after TAVI in reducing the mortality of stroke, the observed discrepancy may stem from the concern among operators that untreated significant CAD could induce ischemia and hemodynamic complications during valve implantation. Consequently, nearly two-thirds of our patients underwent staged PCI prior to TAVI. This finding may not accurately reflect real-world clinical practice. Similarly, Nunes RAB et al. (46) reported that PCI performed prior to TAVI may increase the risk of bleeding and vascular complications. However, in this study, bleeding outcomes were not associated with the timing of PCI, a discrepancy potentially related to differences in the dosages of dual antiplatelet therapy and anticoagulation regimens employed. However, Montalto et al. found that in TAVI patients with concomitant CAD, the incidence of bleeding events during one-year follow-up was significantly higher compared to patients undergoing simultaneous procedures. This observation is inconsistent with our conclusions. Potential explanations include the definition of bleeding as severe hemorrhage in this research, as well as the inclusion of CAD patients with a higher degree of coronary artery stenosis and exceedingly high PCI risk, coupled with the exclusion of patients with a treatment interval of 6 months (38). A meta-analysis by Aarts HM showed (47) that patients who underwent TAVI and preoperative PCI had a higher risk of hematorrhea after 30 days in both short-term and long-term follow-up. Patients undergoing PCI after TAVI exhibit a heightened risk of bleeding, potentially related to the requirement for dual antiplatelet therapy (DAPT). This was also consistent with the study conducted by Tiffany Patterson et al. (40), which attribute the increase in the use of DAPT in patients undergoing PCI to the administration of antithrombotic treatment. Peter Wenaweser's short-term data indicated that (48) the incidence of stroke, bleeding, and vascular complications of the combined therapy was similar compared to that of TAVI alone. It was also concluded that the 30-day mortality rate of staged or synchronous PCI in patients with severe AS who underwent TAVI was comparable. Vikas Singh et al. mainly focused on left main disease (49), and found that TAVIplusPCI during hospitalization could lead to higher cardiovascular mortality rates compared to TAVI alone. However, this article was conducted in a single center, had a very small sample size, only included apical TAVI and lacked a control group.

Venturi G (50), Alberto Alperi (36), and Niels R HolmNiels R Holm (51) et al. found that PCI after TAVI did not significantly increase the incidence of complications in the outcomes of MI and stroke. This finding is inconsistent with our research results, which may be attributable to differences in surgical techniques and material selection. Tomoki Ochiai et al. (52) found that the 2-year all-cause mortality, including cardiovascular event, mortality and bleeding, was similar among the three surgical methods (before, simultaneously, and after). This is contrary to our research findings. The reason for this is that the use of self-expanding TPVR devices for TAVI is not conducive to coronary access and is only performed in PCI before TAVI and TAVIplusPCI. The specific method of PCI was not mentioned in this study, which might be the reason for the increased all-cause mortality and cardiovascular event mortality in PCI after TAVI. Due to the possibility of artificial valve displacement caused by PCI after TAVI operation (53) and the high difficulty of surgery, there is relatively small numbers of studies on PCI after TAVI. The type of valve may affect the feasibility of CA after TAVI, while the use of balloon valvuloplasty for PCI after TAVI is feasible (53, 54). It is worth noting that all PCI after TAVI is performed in patients receiving balloon valvuloplasty (46). Therefore, it may be difficult to broadly apply these findings to all patients undergoing PCI and TAVI.

The advantage of TAVIplusPCI is the elimination of the requirement for specialized CAD diagnosis, thereby potentially reducing the need for additional vascular access and minimizing contrast agent-induced kidney damage (55). The advantage of PCI after TAVI surgery is the use of balloons or self-expanding valves to insert catheters into the coronary ostia after transfemoral TAVI, which can accurately image the aortic root and help avoid placing excessively high self-expanding valves on the ring, thereby reducing the difficulty of entering the coronary ostia (53). However, compared to TAVI alone, the average length of hospital stay for the combination therapy is longer, resulting in higher hospitalization costs (49). It is also unexpectedly found that patients undergoing PCI before TAVI exhibit a higher rate of pacemaker impla。In general, PCI before TAVI is recommended for patients with significant coronary artery disease, acute coronary syndrome, or complex lesions; TAVIplusPCI is recommended in patients with non-complex CAD and low bleeding risk; and PCI after TAVI is recommended in patients with mild/moderate CAD without evidence of ischemia and when TAVI postoperative complications need to be treated first. However, the above recommendations need to be dynamically adjusted based on the following factors, such as operating room conditions, operator proficiency in PCI/TAVI combined operations, patient renal function, vascular access complexity, tolerance to antithrombotic therapy, and lesion interactions like the relationship between aortic valve calcification distribution and coronary artery openings (56–58).

### 4.1 Limitations

Although we have compared different timings of PCI for the first time, there are limitations to this study. Firstly, all the studies included in this review were retrospective cohort studies, which may introduce certain biases. Secondly, the definition of CAD in the included studies is inconsistent. Thirdly, the timings of PCI and the time interval between TAVI and PCI are unclear. Lastly, there are no constraints on the quality of the research articles when we screen and include studies.

## 5 Conclusion

In summary, this research demonstrates that the timing of PCI in patients undergoing TAVI has a significant impact on patient outcomes. Comprehensive analysis suggests that performing PCI prior to TAVI is preferable, as it may effectively reduce all-cause mortality, cardiovascular mortality, and stroke. However, the optimal interval between the two procedures has not yet been established. Future studies should investigate the impact of the interval between PCI and TAVI on patient outcomes.

## Data Availability

The original contributions presented in the study are included in the article/Supplementary Material, further inquiries can be directed to the corresponding author.
