# Supplementary material for: Comparison of different timings of percutaneous coronary intervention in patients with transcatheter aortic valve implantation: a network meta-analysis
Source: Front Cardiovasc Med. 2025 Aug 1;12:1596208. doi: 10.3389/fcvm.2025.1596208 (PMC12354649; doi:10.3389/fcvm.2025.1596208)
Supplement: Supplementary file 1 [file Datasheet1.docx]

**Table S1:** Search strategy.

**PubMed**

| Search number | Query |
| --- | --- |
| 1＃ | "Transcatheter Aortic Valve Replacement"[Mesh] |
| 2＃ | (((TAVI[Title/Abstract]) OR (Transcatheter Aortic Valve Replacement[Title/Abstract])) OR (aortic valve replacement[Title/Abstract])) OR (aortic valve implantation[Title/Abstract]) |
| 3＃ | ("Transcatheter Aortic Valve Replacement"[Mesh]) OR ((((TAVI[Title/Abstract]) OR (Transcatheter Aortic Valve Replacement[Title/Abstract])) OR (aortic valve replacement[Title/Abstract])) OR (aortic valve implantation[Title/Abstract])) |
| 4＃ | "Percutaneous Coronary Intervention"[Mesh] |
| 5＃ | (((PCI[Title/Abstract]) OR (Percutaneous Coronary Intervention[Title/Abstract])) OR (Percutaneous Coronary Revascularization*[Title/Abstract])) OR (Percutaneous Coronary Intervention*[Title/Abstract]) |
| 6＃ | ("Percutaneous Coronary Intervention"[Mesh]) OR ((((PCI[Title/Abstract]) OR (Percutaneous Coronary Intervention[Title/Abstract])) OR (Percutaneous Coronary Revascularization*[Title/Abstract])) OR (Percutaneous Coronary Intervention*[Title/Abstract])) |
| 7＃ | (("Transcatheter Aortic Valve Replacement"[Mesh]) OR ((((TAVI[Title/Abstract]) OR (Transcatheter Aortic Valve Replacement[Title/Abstract])) OR (aortic valve replacement[Title/Abstract])) OR (aortic valve implantation[Title/Abstract]))) AND (("Percutaneous Coronary Intervention"[Mesh]) OR ((((PCI[Title/Abstract]) OR (Percutaneous Coronary Intervention[Title/Abstract])) OR (Percutaneous Coronary Revascularization*[Title/Abstract])) OR (Percutaneous Coronary Intervention*[Title/Abstract]))) |

**Embase**

| Search number | Query |
| --- | --- |
| 1＃ | 'transcatheter aortic valve implantation'/exp |
| 2＃ | tavi:ab,ti OR 'transcatheter aortic valve replacement':ab,ti OR 'aortic valve replacement':ab,ti OR 'aortic valve implantation':ab,ti |
| 3＃ | #1 OR #2 |
| 4＃ | 'percutaneous coronary intervention'/exp |
| 5＃ | pci:ab,ti OR 'percutaneous coronary intervention':ab,ti OR 'percutaneous coronary revascularization*':ab,ti OR 'percutaneous coronary intervention*':ab,ti |
| 6＃ | #4 OR #5 |
| 7＃ | #3 AND #6 |

**Web of science**

| Search number | Query |
| --- | --- |
| 1＃ | TAVI (Topic) or transcatheter aortic valve replacement (Topic) or transcatheter aortic valve implantation (Topic) or percutaneous aortic valve implantation (Topic) or aortic valve implantation (Topic) or aortic valve replacement (Topic) |
| 2＃ | PCI (Topic) or Percutaneous Coronary Intervention (Topic) or Percutaneous Coronary Revascularization* (Topic) or Percutaneous Coronary Intervention* (Topic) |
| 3＃ | #1 and #2 |

**Cochrane**

| Search number | Query |
| --- | --- |
| 1＃ | MeSH descriptor: [Transcatheter Aortic Valve Replacement] explode all trees |
| 2＃ | (TAVI or transcatheter aortic valve implantation or percutaneous aortic valve implantation or aortic valve implantation or aortic valve replacement):ti,ab,kw |
| 3＃ | #1 or #2 |
| 4＃ | MeSH descriptor: [Percutaneous Coronary Intervention] explode all trees |
| 5＃ | (PCI or Percutaneous Coronary Revascularization* or Percutaneous Coronary Intervention*):ti,ab,kw |
| 6＃ | #4 or #5 |
| 7＃ | #3 and #6 |

**Table S2:** Baseline Characteristics of included studies.

| Study | Country | Stype | Intervene | Sample size（m/f） | Age | BMI | follow-up time/month | Ending | Quality scores |
| --- | --- | --- | --- | --- | --- | --- | --- | --- | --- |
| van den Boogert et al.2021 | Netherlands | prospective cohort | TAVI | 427（223/204） | 82.6(78.2±85.4) | 26.5(23.8-30.0) | 132 | ①②④⑤ | 7 |
|  |  |  | PCI-TAVI | 150（67/83） | 82.7(77.8±85.6) | 26.3(24.5-29.0) |  |  |  |
| Abdel-Wahab et al.2012 | Germany | prospective cohort | PCI-TAVI | 48（26/29） | 81±7.06 | 27.87±6.52 | 36 | ①③④⑤ | 7 |
|  |  |  | TAVI | 59（34/36） | 81.1±6.20 | 26.40±4.27 |  |  |  |
| Ochiai et al.2020 | USA | prospective cohort | TAVI-PCI | 143（101/42） | 82.4±7.8 | 27.2±5.4 | 19 | ①③④⑤ | 7 |
|  |  |  | TAVIplusPCI | 77（50/20） | 80.4±9.5 | 26.7±5.9 |  |  |  |
|  |  |  | PCI-TAVI | 38（30/8） | 78.9±10.6 | 27.6±5.9 |  |  |  |
| Beska et al.2021 | UK | retrospective analysis | TAVI | 194（109/85） | 82±7 |  | 48 | ①② | 7 |
|  |  |  | PCI-TAVI | 154（88/66） | 81±7 |  |  |  |  |
| Winter et al.2021 | Austria | prospective cohort | PCI-TAVI | 131(82/49) | 80(77,85) | 27.37(23.86,30.20) | 54 | ①③ | 7 |
|  |  |  | TAVI | 318(150/168) | 81(77,85) | 26.79(23.46,30.07) |  |  |  |
| Lunardi et al.2022 | Italy | retrospective analysis | TAVI-PCI | 46 |  |  | 19 | ①②③⑤ | 7 |
|  |  |  | PCI-TAVI | 98 |  |  |  |  |  |
| Mosleh et al.2023 | Lebanon | retrospective analysis | TAVI | 1364（866/498） | 82.0±7.9 | 29.0±15.9 | 132 | ①③④ | 7 |
|  |  |  | PCI-TAVI | 445（290/155） | 81.5±8.9 | 28.4±7.4 |  |  |  |
| Valvo et al.2023 | USA | retrospective analysis | TAVI | 633（355/278） | 80.92±5.80 | 27.11±4.54 | 168 | ①③④⑤ | 8 |
|  |  |  | TAVIplusPCI | 153（71/82） | 80.71±6.16 | 27.57±4.67 |  |  |  |
| Julius et al.2023 | Germany | retrospective analysis | TAVI | 1733（809/924） | 81.2 （77.0–85.4） | 25.9 （23.4–29.3） |  | ①⑤ | 7 |
|  |  |  | PCI-TAVI | 226（132/94） | 82.1（78.2–85.6） | 25.8 （23.1–28.1） | 72 |  |  |
|  |  |  | TAVIplusPCI | 274（167/107） | 81.8（77.4-86.8） | 25.8（23.4-28.6） |  |  |  |
| Dae et al.2023 | USA | retrospective analysis | TAVI | 885（466/419） | 79.6 | / | 36 | ①②③ | 7 |
|  |  |  | TAVIplusPCI | 885（454/431） | 79.6 | / |  |  |  |
| Dae et al.2024 | USA | retrospective analysis | TAVIplusPCI | 904（479/425） | 80.5 ± 8.1 | / | 36 | ①③④ | 7 |
|  |  |  | PCI-TAVI | 2137（713/1424） | 81.1 ± 7.7 | / |  |  |  |
|  |  |  | TAVI-PCI | 101（61/40） | 79.0 ± 8.4 | / |  |  |  |
| Mohamed et al.2024 | USA | retrospective analysis | TAVI | 278696（148824/129872） | 79.61±8.61 | / | 96 | ①③④ | 7 |
|  |  |  | TAVIplusPCI | 13114（6557/6557） | 80.25±8.73 | / |  |  |  |
| Cristina et al.2023 | Italy | prospective cohort | TAVI | 331（184/147） | 82.5±5.7 | 26.1 ± 4.7 | 22 | ①②④⑤ | 8 |
|  |  |  | PCI-TAVI | 417（290/127） | 80.4±6.7 | - 1. ± 4.4 |  |  |  |

① All-cause mortality; ② Myocardial infarction; ③ Stroke; ④ Bleeding; ⑤ Cardiovascular events
