# Supplementary material for: Comparison of different timings of percutaneous coronary intervention in patients with transcatheter aortic valve implantation: a network meta-analysis
Source: Front Cardiovasc Med. 2025 Aug 1;12:1596208. doi: 10.3389/fcvm.2025.1596208 (PMC12354649; doi:10.3389/fcvm.2025.1596208)
Supplement: Supplementary file 2 [file Datasheet2.pdf]

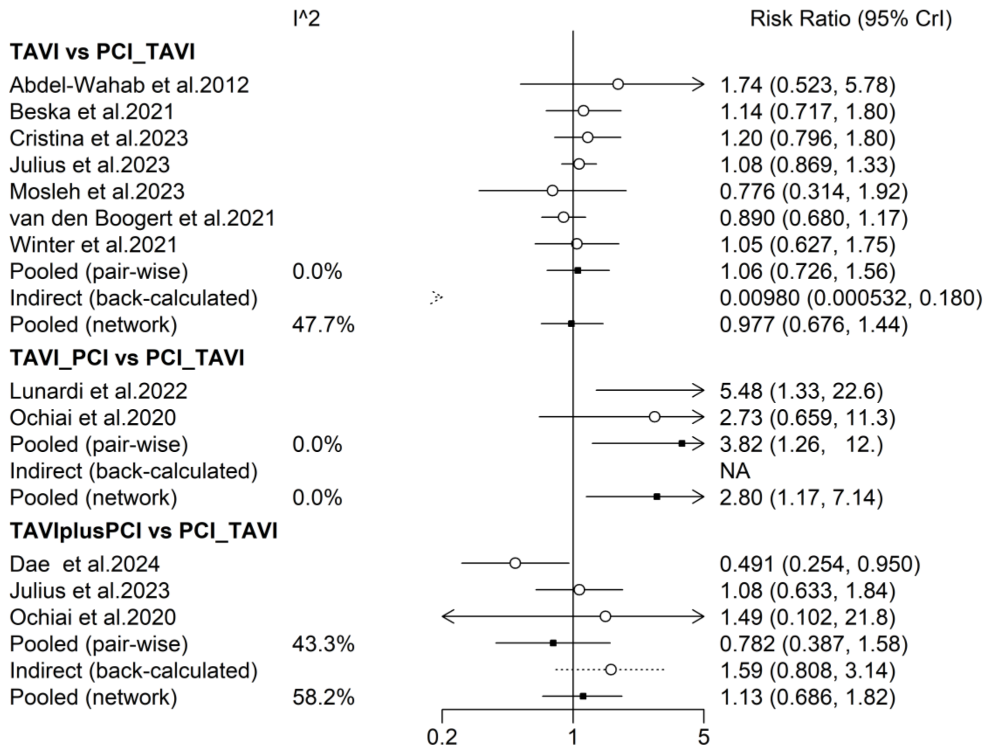

Figure S1 Forest plot of heterogeneity of all-cause mortality.

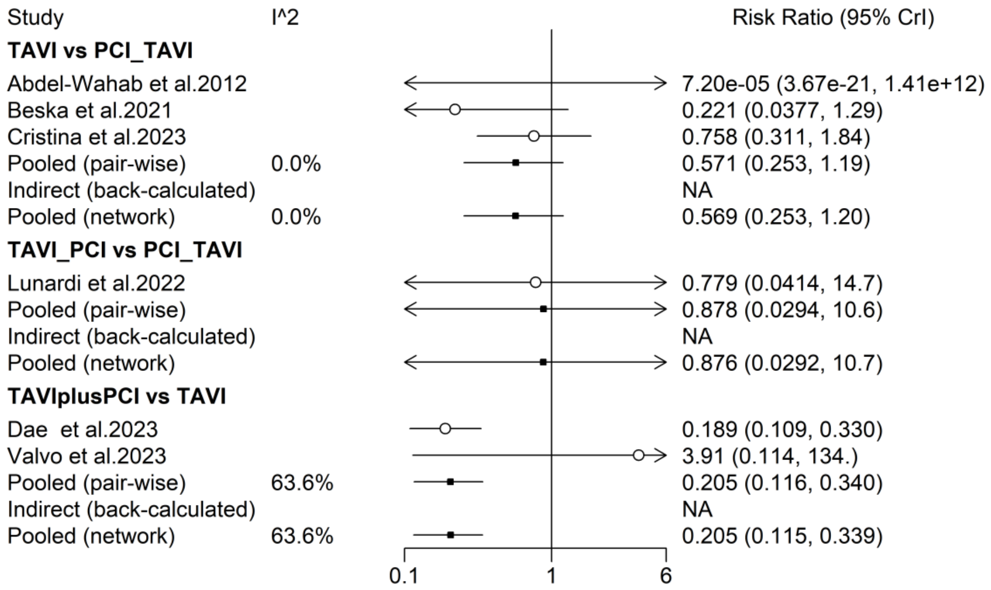

Figure S2 Forest plot of heterogeneity of cardiovascular mortality.

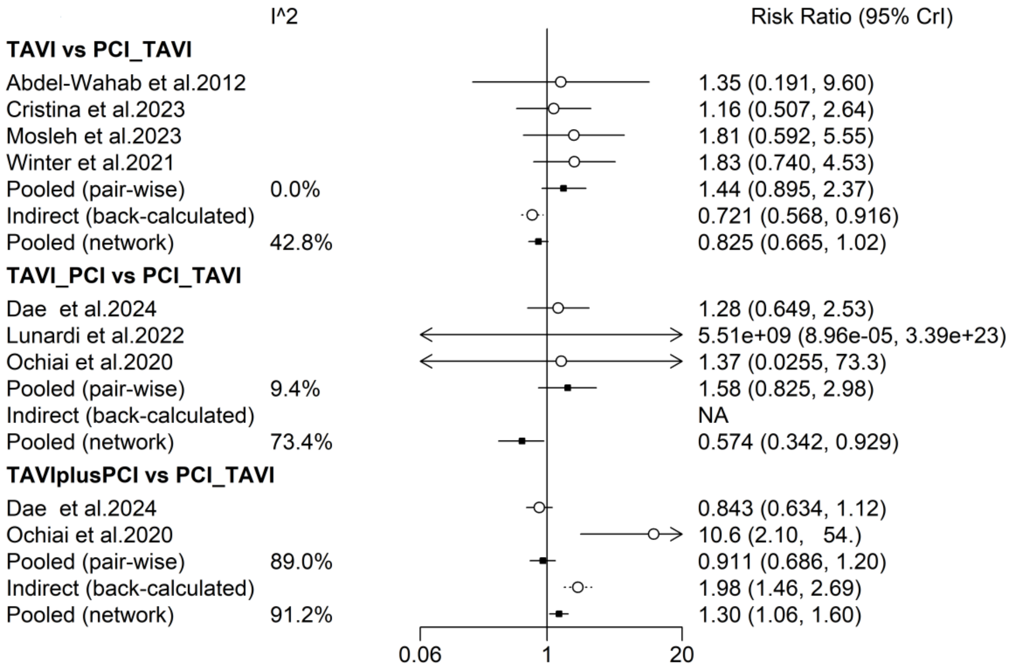

Figure S3 Forest plot of heterogeneity of stroke.

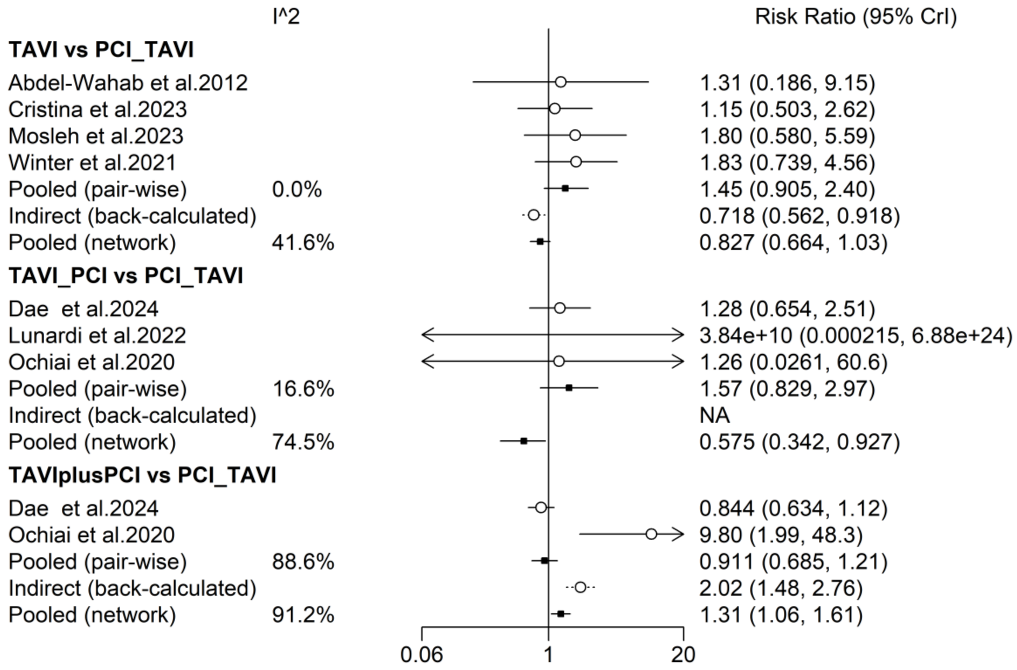

Figure S4 Forest plot of heterogeneity of bleeding.

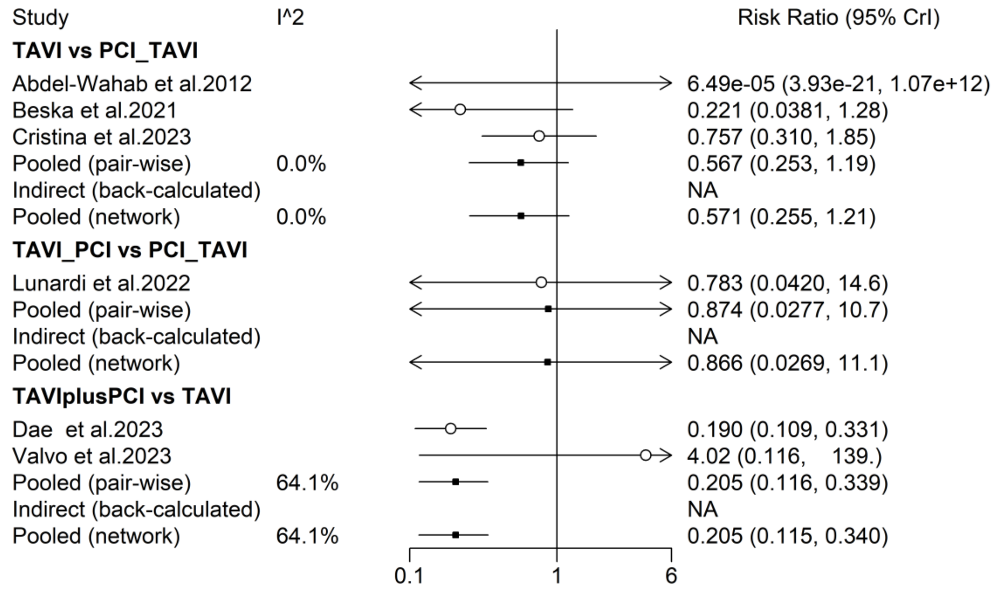

Figure S5 Forest plot of heterogeneity of myocardial infarction.
